# Prediction of IDH and TERT promoter mutations in low-grade glioma from magnetic resonance images using a convolutional neural network

**DOI:** 10.1038/s41598-019-56767-3

**Published:** 2019-12-30

**Authors:** Ryohei Fukuma, Takufumi Yanagisawa, Manabu Kinoshita, Takashi Shinozaki, Hideyuki Arita, Atsushi Kawaguchi, Masamichi Takahashi, Yoshitaka Narita, Yuzo Terakawa, Naohiro Tsuyuguchi, Yoshiko Okita, Masahiro Nonaka, Shusuke Moriuchi, Masatoshi Takagaki, Yasunori Fujimoto, Junya Fukai, Shuichi Izumoto, Kenichi Ishibashi, Yoshikazu Nakajima, Tomoko Shofuda, Daisuke Kanematsu, Ema Yoshioka, Yoshinori Kodama, Masayuki Mano, Kanji Mori, Koichi Ichimura, Yonehiro Kanemura, Haruhiko Kishima

**Affiliations:** 1 Department of Neurosurgery, Graduate School of Medicine, Osaka University, 2-2 Yamadaoka, Suita, Osaka 565-0871 Japan; 2 Department of Neuroinformatics, ATR Computational Neuroscience Laboratories, 2-2-2 Hikaridai, Seika-cho, Kyoto 619-0288 Japan; 3 Institute for Advanced Co-creation studies, Osaka University, 2-2 Yamadaoka, Suita, Osaka 565-0871 Japan; 4 Center for Information and Neural Networks, National Institute of Information and Communications Technology, 1-4 Yamadaoka, Suita, Osaka 565-0871 Japan; 5 Kansai Molecular Diagnosis Network for CNS Tumors, Osaka, 540-0006 Japan; 6 Division of Brain Tumor Translational Research, National Cancer Center Research Institute, Tokyo, 104-0045 Japan; 7 Education and Research Center for Community Medicine, Faculty of Medicine, Saga University, Saga, 849-8501 Japan; 8 Department of Neurosurgery and Neuro-Oncology, National Cancer Center Hospital, Tokyo, 104-0045 Japan; 9 Department of Neurosurgery, Osaka City General Hospital, Osaka, 534-0021 Japan; 10 Department of Neurosurgery, Kindai University Faculty of Medicine, Sayama, 589-8511 Japan; 11 Department of Neurosurgery, National Hospital Organization Osaka National Hospital, Osaka, 540-0006 Japan; 12 Department of Neurosurgery, Kansai Medical University, Hirakata, 573-1191 Japan; 13 Department of Neurosurgery, Rinku General Medical Center, Izumisano, 598-8577 Japan; 14 Department of Neurological Surgery, Wakayama Medical University School of Medicine, Wakayama, 641-0012 Japan; 15 Department of Neurosurgery, Sakai City Medical Center, Sakai, 593-8304 Japan; 16 Division of Stem Cell Research, Department of Biomedical Research and Innovation, Institute for Clinical Research, National Hospital Organization Osaka National Hospital, Osaka, 540-0006 Japan; 17 Division of Regenerative Medicine, Department of Biomedical Research and Innovation, Institute for Clinical Research, National Hospital Organization Osaka National Hospital, Osaka, 540-0006 Japan; 18 Kobe University Graduate School of Medicine, Department of Diagnostic Pathology, 7-5-1 Kusunoki-cho Chuo-ku, Kobe, 650-0017 Japan; 19 Department of Central Laboratory and Surgical Pathology, National Hospital Organization Osaka National Hospital, Osaka, 540-0006 Japan; 20 Department of Neurosurgery, Kansai Rosai Hospital, Amagasaki, 660-8511 Japan; 21 Department of Neurosurgery, Osaka International Cancer Institute, Osaka Prefectural Hospital Organization, Osaka, 541-8567 Japan; 22 Graduate School of Information Science and Technology, Osaka University, 1-5 Yamadaoka, Suita, Osaka 565-0871 Japan

**Keywords:** Biotechnology, Cancer, Computational biology and bioinformatics, Genetics, Neuroscience, Diseases, Medical research, Neurology

## Abstract

Identification of genotypes is crucial for treatment of glioma. Here, we developed a method to predict tumor genotypes using a pretrained convolutional neural network (CNN) from magnetic resonance (MR) images and compared the accuracy to that of a diagnosis based on conventional radiomic features and patient age. Multisite preoperative MR images of 164 patients with grade II/III glioma were grouped by IDH and TERT promoter (pTERT) mutations as follows: (1) IDH wild type, (2) IDH and pTERT co-mutations, (3) IDH mutant and pTERT wild type. We applied a CNN (AlexNet) to four types of MR sequence and obtained the CNN texture features to classify the groups with a linear support vector machine. The classification was also performed using conventional radiomic features and/or patient age. Using all features, we succeeded in classifying patients with an accuracy of 63.1%, which was significantly higher than the accuracy obtained from using either the radiomic features or patient age alone. In particular, prediction of the pTERT mutation was significantly improved by the CNN texture features. In conclusion, the pretrained CNN texture features capture the information of IDH and TERT genotypes in grade II/III gliomas better than the conventional radiomic features.

## Introduction

Identification of IDH1/2 (IDH) mutation and/or TERT promoter (pTERT) mutation is crucial for reaching a correct diagnosis and choosing the most appropriate treatment for patients harboring WHO grade II/III gliomas. In fact, the WHO 2016 brain tumor classification requires both IDH mutation status and 1p/19q chromosomal codeletion status to determine the diagnosis of this tumor^1–4^; 1p/19q chromosomal codeletion status can in some ways be considered equivalent to pTERT mutation status^1,3,5–7^. Although determination of the molecular characteristics of these neoplasms prior to surgical intervention is thought to be beneficial for patient care, this information can currently be obtained only after surgically removing the tissue. Several previous studies challenged this issue by pursuing preoperative molecular characterization of WHO grade II/III gliomas using magnetic resonance imaging (MRI) and radiomic analysis^8–12^. This scientific objective was further pursued by attempting to boost the diagnostic accuracy through machine learning algorithms such as a deep neural network (DNN)^13,14^ or convolutional neural network (CNN)^15,16^. Indeed, a recent study by Chang *et al*. applied DNN to MR images of WHO grade II to IV gliomas and demonstrated that IDH1/2 mutations can be successfully classified from MR images^17^. However, the question remains as to whether a DNN or CNN can enhance diagnostic accuracy in the radiogenomics of gliomas compared to conventional radiomic analysis. The effect on the diagnostic accuracy of adding clinical information, such as patient age, is also not completely understood.

**Figure 1:**
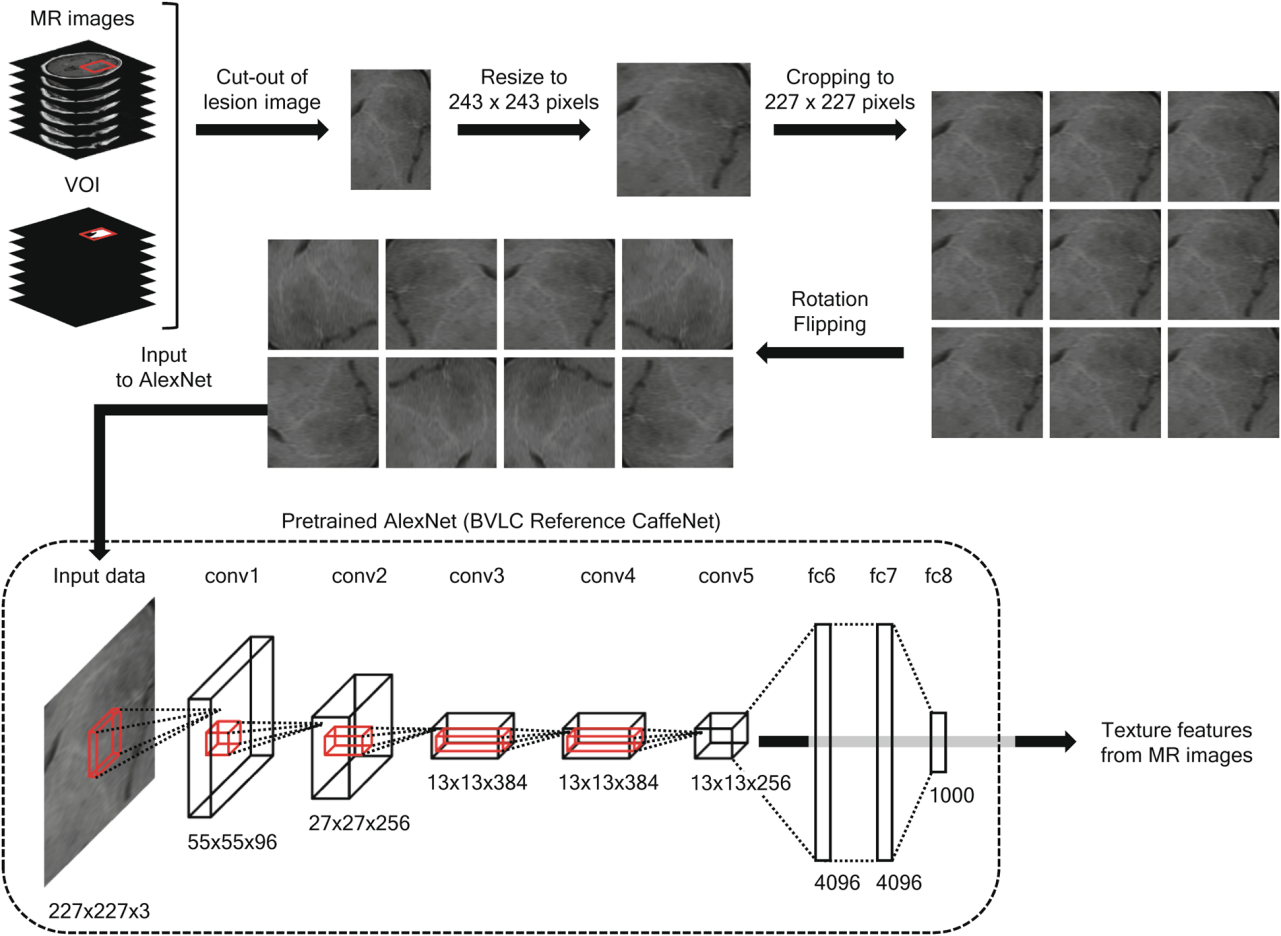
Feature extraction by AlexNet. The lesion image was cut out using a VOI and resized to 243 × 243 pixels. The image was cropped to 227 × 227 pixels with a shift of ±8 pixels and rotated/flipped for data augmentation. The augmented image was input to the pretrained AlexNet to acquire the texture features for classification.

In this study, we sought to reveal a positive impact of sophisticated machine learning algorithms on the radiomic diagnostic accuracy of predicting the molecular characteristics of WHO grade II/III gliomas. More specifically, the diagnostic accuracy concerning the molecular characteristics of WHO grade II/III gliomas was compared between a conventional radiomic image texture extraction technique and an automatic image analysis technique using the CNN trained by natural scenes, the AlexNet^18^. The impact of adding clinical information to model the prediction of molecular characteristics for this neoplasm was also evaluated (Fig. 1).

## Results

### Optimization of a convolutional neural network for glioma feature extraction from a magnetic resonance image

**Figure 2:**
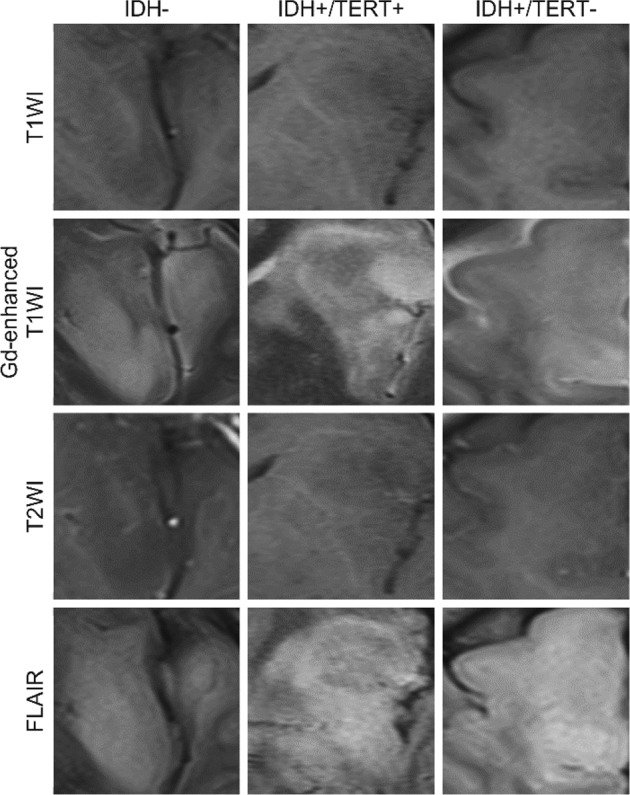
Example of input images. Representative lesion images of each sequence for each molecular subtype of WHO grade II/III gliomas were shown.

**Figure 3:**
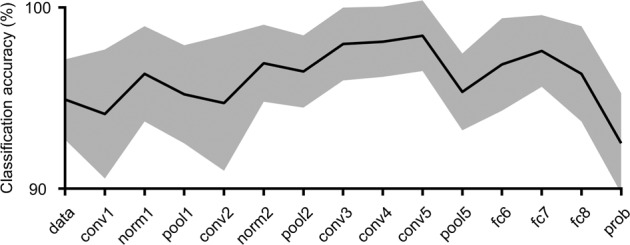
Classification accuracy of lesions from normal tissue. The average classification accuracies in all folds of cross-validation are shown with 95% confidence intervals.

The CNN (AlexNet) layer was first tuned for glioma MR image feature extraction. In order to extract texture features (Fig. 1), MR images were normalized in their intensity, and from the slice locating center of the lesion, a lesion image was cut out at the boundary of the lesion for each patient and for each sequence (Fig. 2): T1-weighted (T1W) imaging, T2-weighted (T2W) imaging, gadolinium-enhanced T1-weighted (GdT1W) imaging, and fluid-attenuated inversion recovery (FLAIR) imaging. Texture features of the lesion image were acquired from neurons from each layer between data input to probability output. Linear support vector machine (SVM) models were trained to distinguish between brain tumor lesions and normal tissues, using the texture features of images of brain tumor lesions and images of normal tissue. Figure 3 shows the classification accuracies for each CNN layer using 10-fold cross-validation. The classification accuracy between the lesion and normal tissue was highest when the texture features from conv5 were used to train the SVM classifier (98.4 ± 1.9% (0.999 ± 0.003) [mean ± 95% confidence interval (mean ± 95% confidence interval of area under the precision-recall curve)]). As a result, the texture features from AlexNet conv5 were further applied in this study.

### Comparison of molecular diagnostic accuracy of WHO grade II/III gliomas between radiomics and the convolutional neural network

**Figure 4:**
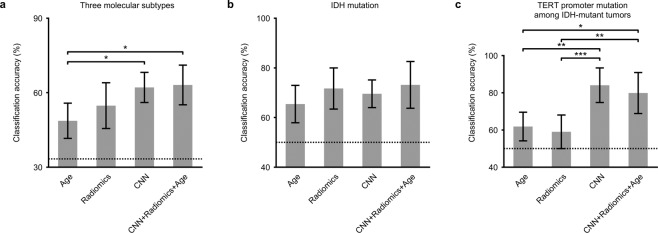
Classification accuracy of genotype status for different features. (**a**–**c**) Each bar shows the averaged classification accuracy of three molecular subtypes consisting of 1) IDH wild type, 2) IDH and pTERT comutated, and 3) IDH mutant and pTERT wild type (**a**), the IDH mutation alone (**b**), or the pTERT mutation alone (**c**). The label of each bar denotes the features used for the classification: Age, patient age; Radiomics, conventional radiomic features from MR images with three location parameters; CNN, features extracted by AlexNet; CNN + Radiomics + Age, all of these features. The average was calculated from the accuracies, which were balanced among classes, for each test dataset in 10-fold nested cross-validation. Error bars show 95% confidence intervals of the classification accuracy. Dotted lines denote chance level. *p < 0.05, **p < 0.01, and ***p < *0.001 significant difference among different features (one-way ANOVA with a Tukey-Kramer post hoc test)*.

Combinations of the following sets of information were fed to SVMs in an attempt to train an SVM classifier for molecular subtypes of WHO grade II/III gliomas: (1) the age of the patient, (2) 61 conventional radiomic features based on MRI^8^ and 3 location parameters (radiomic features), and (3) 4000 texture features selected from AlexNet conv5 analysis of T1W, T2W, GdT1W, and FLAIR images (CNN features). The three molecular subtypes consisted of (1) IDH wild type (*n* = 56), (2) IDH and pTERT comutations (*n* = 54), and (3) IDH mutant and pTERT wild type (*n* = 54). The molecular subtypes were successfully classified using the following information: patient age, radiomic features and CNN feature; classification accuracies, balanced among classes, were significantly higher than chance (*p* < 0.01, one-tailed Welch’s *t*-test; Fig. 4a). Classification accuracies using the information differed significantly according to the combination of information (age: 48.7 ± 7.1% (0.486 ± 0.063); conventional radiomic features with 3 location parameters: 54.8 ± 9.2% (0.604 ± 0.081); CNN features: 62.1 ± 6.0% (0.636 ± 0.071); all features: 63.1 ± 8.0% (0.649 ± 0.090); *p* = 0.0446, *F*(3,36) = 3.9963, one-way ANOVA, Bonferroni corrected). Among the three types of features, the accuracy using the CNN features was the highest and significantly higher than that using patient age (*p* = 0.0386, post hoc Tukey-Kramer test, Fig. 4a). Notably, the accuracies were not significantly different between the analysis using the conventional radiomic features and patient age; combination of these features did not improve the classification accuracy (54.3 ± 6.8% (0.613 ± 0.055)). Moreover, the classification accuracy was highest when all of the features were used together for the classification, although the accuracy using all features was not significantly different from that using CNN features alone (Fig. 4a). It therefore appears that among the three types of features, the CNN-based features were the most informative in classifying the molecular subtypes of WHO grade II/III gliomas.

### Comparison of classification accuracy for IDH mutation of WHO grade II/III gliomas

To clarify how the IDH mutation and pTERT mutation affect classification accuracy, we classified the IDH mutation of WHO grade II/III gliomas. The balanced classification accuracies for IDH mutation were significantly higher than that expected by chance for each feature (*p* < 0.01, one-tailed Welch’s *t*-test, Fig. 4b). Although the balanced classification accuracies were highest with all the features combined (age: 65.4 ± 7.5% (0.558 ± 0.090); conventional radiomic features with 3 location parameters: 71.7 ± 8.3% (0.718 ± 0.139); CNN features: 69.6 ± 5.6% (0.619 ± 0.132); all features: 73.1 ± 9.4% (0.699 ± 0.145); Fig. 4b), a statistically significant difference was not observed among these accuracies (*p* = 1.2940, *F*(3,36) = 0.9402, one-way ANOVA, Bonferroni corrected). These results suggest that conventional radiomic features and CNN-based texture features extract similar information from MR images concerning IDH mutations in WHO grade II/III gliomas.

### Comparison of classification accuracy for TERT promoter mutation in IDH-mutated WHO grade II/III gliomas

Finally, classification of the pTERT mutation in IDH-mutated WHO grade II/III gliomas was pursued. The pTERT mutation was successfully classified using each of the features with an accuracy greater than chance (*p* < 0.01, one-tailed Welch’s *t*-test, Fig. 4c) except using conventional radiomic features with 3 location parameters (*p* = 0.257, *t*(17.4) = 0.6674). Classification accuracies, balanced among classes, differed significantly based on the features used (age: 61.8 ± 7.7% (0.621 ± 0.122); conventional radiomic features with 3 location parameters: 59.0 ± 9.0% (0.656 ± 0.113); CNN features: 84.0 ± 9.3% (0.868 ± 0.099); all features: 79.8 ± 11.0% (0.861 ± 0.116); *p* = 0.0003, *F*(3,36) =  9.3406, one-way ANOVA, Bonferroni corrected). The classification accuracies using CNN base texture features and all features together were both significantly higher than those using patient age alone or conventional radiomic features with 3 location parameters (patient age vs. CNN base texture features, *p* = 0.0028; patient age vs. all features, *p* = 0.0192; conventional radiomic features with 3 location parameters vs. CNN base texture features, *p* = 0.0007; conventional radiomic features with 3 location parameters vs. all features *p* = 0.0053, Tukey-Kramer post hoc test). These results suggest that the CNN features succeeded in capturing some of the characteristics of the pTERT mutation that were not captured by conventional radiomic features and patient age.

## Discussion

Noninvasive molecular characterization of neoplasms via radiomics or convolutional neural network (CNN) analysis of radiographic images is a rapidly expanding research field. It is believed that the development of this type of technology could be a substitute for a large portion of – if not all – invasive procedures involved in acquiring information concerning the fundamental nature(s) of neoplastic disease. This diagnostic concept has been widely explored for lung cancers, breast cancers and, recently, gliomas, for all of which genetic characterization of the tumor is crucial for determining an accurate diagnosis and selecting the most appropriate treatment strategy^19–29^. Historically, this proof-of-concept strategy was initially tested by introducing the idea of ‘radiomics’ – a coinage based on ‘genomics’ and ‘proteomics’ – that consists of handling numerous texture features of radiographic images to construct an objective-driven prediction model^30^. Combined with automatic segmentation of tumor^31^, these radiomic approaches may be a powerful tool in determining diagnosis and treatment in the future.

Regarding WHO grade II/III gliomas, several attempts have shown that this approach holds promise for identifying genetic alterations in gliomas. In fact, IDH mutations can be readily predicted with an accuracy greater than 80%. These studies have also highlighted the limitation of this approach. Notably, pTERT mutation or 1p/19q codeletion, either of which are crucial information for discriminating oligodendroglial tumors from astrocytic tumors, cannot be predicted within a reasonable accuracy. The introduction of the CNN was thought to enable feature extraction of images that were overlooked by handcrafted feature extraction used in radiomics. The current investigation indeed reveals that a CNN can provide valuable texture information, especially for discriminating the pTERT mutation, a task that radiomics alone cannot achieve successfully. Indeed, Fig. 4c shows that the CNN enabled pTERT mutation classification accuracy to jump from 59% to 84% when the analyzed cohort was confined to IDH mutant tumors. The three-class classification accuracy also improved from 55% to 63% with the aid of the CNN. These observations strongly suggest that CNN analysis is superior to radiomics in detecting some types of genetic alterations. It is also noteworthy that there are genetic alterations that CNN analysis has no advantage in detecting compared to radiomics. As in this investigation, CNNs usually analyze two-dimensional images, while radiomics performs three-dimensional data acquisition, and this is a theoretical difference in which radiomics may be superior to CNN analysis in some cases. The conclusion that can be drawn from this phenomenon is that the use of, and results from, a CNN should always be critically and quantitatively evaluated to ensure its superiority over conventional radiomics.

The CNN used in this investigation was a natural scene pretrained CNN. It is possible that training the CNN with glioma images could improve genetic alteration classification accuracy, and this scientific question should be pursued in future investigations. Indeed, a similar approach has been reported, focusing on the IDH mutation status of WHO grade II–IV gliomas^17^. The scientific and clinical significance of the current research is that the investigated cohort is tightly restricted to WHO grade II/III gliomas. Because age alone has a great impact on classifying IDH mutation status, clinicians are already aware that IDH wild-type and mutant tumors exhibit completely different clinical presentations. More specifically, grade IV gliomas (glioblastomas) are mostly IDH wild-type tumors and are prone to showing contrast enhancement and elderly age distribution. IDH mutant tumors are mainly grade II or III gliomas and are less likely to show massive contrast enhancement and tend to occur in younger patients. If the cohort of the analysis, regardless of the technique used for image analysis (i.e., radiomics-based or CNN-based analysis), was performed without carefully discriminating the two, IDH mutation can be easily classified by age or contrast enhancement of the tumor alone, which adds no value in clinical practice, as tumor contrast enhancement has long been considered a hallmark of glioblastoma (i.e., IDH wild-type tumor) in many cases. The real clinical value of this technique appears when it achieves accurate diagnosis in situations where conventional knowledge is unable to do so. The current investigation took this clinical question seriously and raised the bar for predicting molecular alterations in gliomas by restricting the analyzed cohort to WHO grade II/III gliomas and was able to prove not only that IDH mutation can be classified at a reasonable accuracy but also that the classification accuracy of the pTERT mutation can be improved by a CNN, which warrants future investigations for training the CNN itself with a WHO grade II/III glioma-restricted cohort.

## Materials and Methods

### Patient cohorts

This study adhered to the Declaration of Helsinki and was performed in accordance with protocols approved by the internal ethical review boards of the Osaka International Cancer Institute (No. 1306055036), Osaka University Graduate School of Medicine (No. 13244), and all collaborating institutes. All patients were enrolled after obtaining written informed consent.

The inclusion criteria was as follows: (1) 20 years of age or older; (2) frozen or fresh tissue available for genomic analysis; (3) pre-operative MRI available for radiomic analysis; and (4) local diagnosis of lower grade (WHO grade II/III) glioma based on the fourth edition of the WHO classification^32^. A total of 199 cases from 11 institutions were collected and included for analysis. MR images were acquired using either a 1.5 T or 3.0 T MRI scanner according to the protocols at each institution with a wide variety of MR venders. A total of 164 datasets comprising T1W, T2W, FLAIR, and GdT1W images, were send for further analysis. All of the images were acquired in the axial plane.

### Diagnosis, central pathology and genetic analysis

All cases analyzed in this study were the same as the cases that we previously reported^8^. A senior neuropathologist performed central pathological reviews of all cases. Integrated diagnosis was made based on the microscopic histological diagnosis and the status of *IDH*1/2 and 1p/19q copy number in compliance with the CNS WHO2016^33^. The following two laboratories conducted the genetic analysis: the Osaka National Hospital in Osaka, Japan, and the National Cancer Center Research Institute in Tokyo, Japan. Hotspot mutations of *IDH*1/2 (codon 132 of *IDH*1 and codon 172 of *IDH*2) and the *TERT* promoter (pTERT, termed C228 and C250) were assessed by Sanger sequencing and/or pyrosequencing at either laboratory (detailed information can be found in previous publications)^6,8^. This study included 56 samples with IDH wild type, 54 samples with IDH- and pTERT-mutated, and 54 samples with IDH-mutated but pTERT wild type, of which 31, 34, and 25 samples were acquired by 1.5 T scanner, respectively. There was no significant relationship between the molecular subtypes and the MRI scanners (1.5 T (*n* = 90), or 3.0 T (*n* = 74)) (*p* = 0.219, *X*^2^(2, *n* = 164) = 3.0367, chi-squared test).

### Tumor segmentation and conventional radiomic analysis

The radiomic analyses (radiomics) were performed using the method reported in our previous study^8^. An in-house-developed MATLAB-based (Mathworks, Natick, MA) image analysis software was used in combination with the Oxford Centre for Functional MRI of the Brain (FMRIB) Linear Image Registration Tool (FLIRT) provided by the FMRIB Software Library (FSL)^34–36^. First, MRIConvert (University of Oregon Lewis Center for Neuroimaging: http://lcni.uoregon.edu/~jolinda/MRIConvert/) converted all Digital Imaging and Communications in Medicine (DICOM) format images to Neuroimaging Informatics Technology Initiative (NIfTI) format. All NIfTI files were sent to intensity normalization processing. Voxels that were in the top 0.1% in intensity histogram were considered high signal noise and therefore deleted, and the remaining 99.9% were reallocated to 256 grayscale among noncontrast T1W, contrast-enhanced GdT1W, and FLAIR, but not for T2W images. For T2W images, 100% of the data range was reallocated to 256 grayscale. T2Edge images were constructed by applying a Prewitt filter to T2W images. Gdzscore images were also constructed by performing a voxelwise contrast enhancement calculation using noncontrast and contrast-enhanced GdT1W images. Detailed methods can be found in Table S1. Tumors were segmented by an experienced surgical neuro-oncologist manually tracing high-intensity lesions on T2W images in three dimensions. Next, T2W images were registered to the standard MNI152 space, and the segmented tumors were mapped on the MNI152 coordinate system using the conversion matrix of T2W to MNI152. FSL-FLIRT was used for image registration with 12 degrees of freedom via mutual information algorithm.

Furthermore, all image sequences obtained from a single subject were coregistered with each other, thus enabling the created T2W-based lesions to be applied to different sequences. Three different aspects of the tumor were measured: (1) histogram-based first-order texture; (2) shape; and (3) location (Table S1). Lesion location information was also calculated based on the weight center of the VOI in MNI152 space, which consisted of 3 values corresponding to the x, y, z coordinates of the point located within MNI152.

### Image preprocessing

The intensity of each MR image was normalized among the entire slices so that the 2.5th and 97.5th percentiles of the voxels became the minimum and maximum values, respectively. The VOI of the lesion on T2W images was coregistered to the T1W and FLAIR images to identify the tumor area within each of these images. GdT1W images were resliced to the T1WI space to use the VOI created for the T1W images.

### CNN feature extraction

For each MRI sequence, an axial slice that contained the lesion was selected at first to extract CNN texture features using the VOI of the lesion coregistered to the sequence. On the center slice of the VOI in the axial direction, an image was cut out by the boundary box of the VOI (lesion image) and resized to 243 × 243 pixels (because each lesion was a different size). To artificially enlarge the dataset^18^, data augmentation was performed by cropping, flipping, and rotating the lesion image (Fig. 1). For the cropping, nine images of 227 × 227 pixels were cropped from a single resized lesion image of 243 × 243 pixels with shift of 0 or ±8 pixels in the x/y direction from its center. The cropped images were then rotated by 0, 90, 180, or 270 degrees and either horizontally flipped or not (resulting in 72 images) prior to inputting them to the pretrained BVLC CaffeNet running on Chainer^37^. The BVLC CaffeNet was AlexNet^18^ with a minor variation developed by Berkeley Vision and Learning Center, who also trained the weights of the network using the ILSVRC-2012 ImageNet dataset on Caffe^38^. Outputs of neurons in each layer other than the ReLU and dropout layers were extracted as CNN texture features.

To extract texture features of normal tissue, the VOIs modeled on the T2W images were flipped in the sagittal plane and allocated manually in another hemisphere to define a normal tissue region. The allocated normal tissue region on the T2W images was coregistered to the MR images of different sequences; in exactly the same way, to extract the texture features of the lesion, the normal image was cut out using the coregistered normal tissue region as the VOI and augmented before extracting the texture features. During this process, the normal tissue region could not be allocated without overlap between the lesion image and the normal image in 37 patients, so these patients were excluded from the analysis decoding the lesion from normal tissue.

### Classification

To select the appropriate layer for decoding the genotype status for each layer of the CNN, classification analysis between the lesion and normal tissue was performed in 127 patients using linear SVMs. For each layer, 1000 neurons were first randomly selected to apply SVM directly, and the texture features from these neurons were concatenated among the MR images (resulting in 4000 features) to train the SVMs. A 10-fold nested cross validation was adopted. Each test dataset of the outer cross-validation was classified by a linear SVM that was trained using the training dataset of the outer cross-validation, with a cost parameter optimized by another cross-validation within the training dataset. The division of the dataset into the test dataset and training dataset was controlled so that the two datasets did not contain training samples from the same patient. The classification accuracy accounted for only the decoding results using the texture feature from the center of the lesion image or the normal image, with neither rotation nor horizontal flip applied. For each test dataset of the outer cross-validation, area under the precision-recall curve (AUPRC) was calculated using distances of the samples to the separating hyperplane.

The genotype status of each patient was classified using the SVM for the following three conditions: three molecular subtypes (IDH wild type, IDH and pTERT mutations, or IDH mutation with pTERT wild type; totally, *n* = 164); IDH (mutation or wild type; *n* = 164); and pTERT (mutation or wild type; *n* = 108). Again, 10-fold nested cross validation was applied in the same manner as in the classification analysis between the lesion and normal tissue. For further analysis, the division of the dataset was kept the same during the classification analyses for the same status of genotypes but with different features. In the analysis using the CNN texture features, the number of features for building the SVM was reduced to 4000 by selecting the features with the highest *F*-statistics, which were calculated only among the training dataset. Moreover, classification accuracy using the CNN texture features again accounted for only the classification using features extracted from the center of the lesion image, with neither rotation nor horizontal flip. Each classification accuracy was balanced among classes, so that it was not affected by an imbalanced number of samples among classes. Finally, AUPRCs were again calculated. In the classification analysis of the three molecular subtypes, distance to the hyperplane that separated the target subtype from the other two subtypes was used to calculate the AUPRC; the AUPRCs of the three subtypes were then averaged within the same test dataset of the outer cross-validation.

### Statistics

For each decoding condition for the three molecular subtypes, classification analysis was performed four times using the following features: patient age, 64 radiomic features (including lesion locations), CNN features, and the combination of these three features. The classification accuracies of the 10-fold validations were compared using one-way analysis of variance (ANOVA) with a Tukey-Kramer post hoc test. Bonferroni correction was applied to the *p*-value of the ANOVA for the three classifications of genotype status. For each classification analysis, the significance of the classification accuracy was tested by a one-tailed Welch’s *t*-test compared to the classification accuracy expected by chance. The chance classification accuracy was estimated by another classification analysis using the age feature with a shuffled label for the genotype status.

## Supplementary information

Supplementary information.

## Data Availability

The data are not publicly available because they contain information that could compromise research participants’ privacy and/or consent.
