# Supplementary material for: Prediction of IDH and TERT promoter mutations in low-grade glioma from magnetic resonance images using a convolutional neural network
Source: Sci Rep. 2019 Dec 30;9:20311. doi: 10.1038/s41598-019-56767-3 (PMC6937237; doi:10.1038/s41598-019-56767-3)
Supplement: Supplementary file 1 — Supplementary information. [file 41598_2019_56767_MOESM1_ESM.pdf]

Prediction of IDH and TERT promoter mutations in low-grade glioma from magnetic resonance images using a convolutional neural network

Ryohei Fukuma[1-2], Takufumi Yanagisawa[1–3]*, Manabu Kinoshita[1]*, Takashi Shinozaki[4], Hideyuki Arita[1,5,6], Atsushi Kawaguchi[7], Masamichi Takahashi[8], Yoshitaka Narita[8], Yuzo Terakawa[5,9], Naohiro Tsuyuguchi[5,9,10], Yoshiko Okita[21], Masahiro Nonaka[5,11,12], Shusuke Moriuchi[5,11,13], Masatoshi Takagaki[1,5], Yasunori Fujimoto[1,5], Junya Fukai[5,14], Shuichi Izumoto[5,10], Kenichi Ishibashi[5,9], Yoshikazu Nakajima[5,15], Tomoko Shofuda[5,16], Daisuke Kanematsu[5,17], Ema Yoshioka[5,16], Yoshinori Kodama[18], Masayuki Mano[5,19], Kanji Mori[5,20], Koichi Ichimura[6], Yonehiro Kanemura[5,17], Haruhiko Kishima[1]

[1] Department of Neurosurgery, Graduate School of Medicine, Osaka University, 2-2 Yamadaoka, Suita, Osaka 565-0871, Japan

[2] Department of Neuroinformatics, ATR Computational Neuroscience Laboratories, 2-2-2 Hikaridai, Seika-cho, Kyoto 619-0288, Japan

[3] Institute of Advanced Cocreation studies, Osaka University, 2-2 Yamadaoka, Suita, Osaka 565-0871, Japan

[4] CiNet, National Institute of Information and Communications Technology, Japan.

[5] Kansai Molecular Diagnosis Network for CNS Tumors, Osaka, 540-0006, Japan

[6] Division of Brain Tumor Translational Research, National Cancer Center Research Institute, Tokyo, 104-0045, Japan

[7] Education and Research Center for Community Medicine, Faculty of Medicine, Saga University, Saga, 849-8501, Japan

[8] Department of Neurosurgery and Neuro-Oncology, National Cancer Center Hospital, Tokyo, 104-0045, Japan

[9] Department of Neurosurgery, Osaka City University Graduate School of Medicine, Osaka, 545-0051, Japan

[10] Department of Neurosurgery, Kindai University Faculty of Medicine, Sayama, 589-8511, Japan

[11] Department of Neurosurgery, National Hospital Organization Osaka National Hospital, Osaka, 540-0006, Japan

[12] Department of Neurosurgery, Kansai Medical University, Hirakata, 573-1191, Japan

[13] Department of Neurosurgery, Rinku General Medical Center, Izumisano, 598-8577, Japan

[14] Department of Neurological Surgery, Wakayama Medical University School of Medicine, Wakayama, 641-0012, Japan

[15] Department of Neurosurgery, Sakai City Medical Center, Sakai, 593-8304, Japan

[16] Division of Stem Cell Research, Department of Biomedical Research and Innovation, Institute for Clinical Research, National Hospital Organization Osaka National Hospital, Osaka, 540-0006, Japan

[17] Division of Regenerative Medicine, Department of Biomedical Research and Innovation, Institute for Clinical Research, National Hospital Organization Osaka National Hospital, Osaka, 540-0006, Japan

[18] Kobe University Graduate School of Medicine, Department of Diagnostic Pathology, 7-5-1 Kusunoki-cho Chuo-ku Kobe 650-0017, Japan

[19] Department of Central Laboratory and Surgical Pathology, National Hospital Organization Osaka National Hospital, Osaka, 540-0006, Japan

[20] Department of Neurosurgery, Kansai Rosai Hospital, Amagasaki, 660-8511, Japan

[21] Department of Neurosurgery, Osaka International Cancer Institute, Osaka Prefectural Hospital Organization, 541-8567, Japan

Corresponding author:

Takufumi Yanagisawa, M.D., Ph.D.,

Institute of Cocreation studies, Osaka University, 2-2 Yamadaoka, Suita, Osaka 565-0871,

Japan, Phone: +81-6-6879-3652, Fax: +81-6-6879-3659,

E-mail: tyanagisawa@nsurg.med.osaka-u.ac.jp

Manabu Kinoshita, M.D., Ph.D.,

Department of Neurosurgery, Osaka University, 2-2 Yamadaoka, Suita, Osaka 565-0871,

Japan, Phone: +81-6-6879-3652, Fax: +81-6-6879-3659,

E-mail: mail@manabukinoshita.com

Radiomic designation code	Contents of values
Histogram and texture parameters	
T2_Mean	Mean of VOI in 256-leveled T2-weighted image.
T2_SD	Standard deviation of VOI in 256-leveled T2-weighted image.
T2_Max	Maximum of VOI in 256-leveled T2-weighted image.
T2_Min	Minimum of VOI in 256-leveled T2-weighted image.
T2_Median	Median of VOI in 256-leveled T2-weighted image.
T2_Mode	Mode of VOI in 256-leveled T2-weighted image.
T2_Entropy	Entropy[1)] of VOI in 256-leveled T2-weighted image.
T2Edge_Mean	Mean of the rim of VOI in Prewitt filtered[2)] 256-leveled T2-weighted image.
T2Edge_SD	Standard deviation of the rim of VOI in Prewitt filtered[2)] 256-leveled T2-weighted image.
T2Edge_Max	Maximum of the rim of VOI in Prewitt filtered[2)] 256-leveled T2-weighted image.
T2Edge_Min	Minimum of the rim of VOI in Prewitt filtered[2)] 256-leveled T2-weighted image.
T2Edge_Median	Median of the rim of VOI in Prewitt filtered[2)] 256-leveled T2-weighted image.
T2Edge_Mode	Mode of the rim of VOI in Prewitt filtered[2)] 256-leveled T2-weighted image.
T2Edge_Entropy	Entropy[1)] of the rim of VOI in Prewitt filtered[2)] 256-leveled T2-weighted image.
T1_Mean	Mean of VOI in 256-leveled T1-weighted image.
T1_SD	Standard deviation of VOI in 256-leveled T1-weighted image.
T1_Max	Maximum of VOI in 256-leveled T1-weighted image.
T1_Min	Minimum of VOI in 256-leveled T1-weighted image.
T1_Median	Median of VOI in 256-leveled T1-weighted image.
T1_Mode	Mode of VOI in 256-leveled T1-weighted image.
T1_Entropy	Entropy[1)] of VOI in 256-leveled T1-weighted image.
T1Gd_Mean	Mean of VOI in 256-leveled Gadolinium enhanced T1-weighted image.
T1Gd_SD	Standard deviation of VOI in 256-leveled Gadolinium enhanced T1-weighted image.
T1Gd_Max	Maximum of VOI in 256-leveled Gadolinium enhanced T1-weighted image.
T1Gd_Min	Minimum of VOI in 256-leveled Gadolinium enhanced T1-weighted image.
T1Gd_Median	Median of VOI in 256-leveled Gadolinium enhanced T1-weighted image.
T1Gd_Mode	Mode of VOI in 256-leveled Gadolinium enhanced T1-weighted image.
T1Gd_Entropy	Entropy[1)] of VOI in 256-leveled Gadolinium enhanced T1-weighted image.
Gdzscore_Mean	Mean of VOI in Gdzscore[3] image.
Gdzscore_SD	Standard deviation of VOI in Gdzscore[3] image.
Gdzscore_Max	Maximum of VOI in Gdzscore[3] image.

Gdzscore_Min	Minimum of VOI in Gdzscore[3] image.
Gdzscore_Median	Median of VOI in Gdzscore[3] image.
Gdzscore_Mode	Mode of VOI in Gdzscore[3] image.
Gdzscore_Entropy	Entropy[1)] of VOI in Gdzscore[3] image.
Gdzscore_area_of_Gd	Ratio of volume with Gdzscore>2.0 within the VOI.
FLAIR_Mean	Mean of VOI in 256-leveled FLAIR.
FLAIR_SD	Standard deviation of VOI in 256-leveled FLAIR.
FLAIR_Max	Maximum of VOI in 256-leveled FLAIR.
FLAIR_Min	Minimum of VOI in 256-leveled FLAIR.
FLAIR_Median	Median of VOI in 256-leveled FLAIR.
FLAIR_Mode	Mode of VOI in 256-leveled FLAIR.
FLAIR_Entropy	Entropy[1)] of VOI in 256-leveled FLAIR.
Shape related parameters	
totalsurfarea	Total surface area (*A*) of the VOI.
totalvol	Total volume (*V*) of the VOI.
compactness01	Value calculated by the following equation; $compactness1 = \frac{V}{\sqrt{\pi} * A^{\frac{2}{3}}}$
compactness02	Value calculated by the following equation; $compactness2 = 36\pi \frac{A^2}{V^3}$
spherical_disproportion	Value calculated by the following equation; $spherical\_disproportion = \frac{A}{4\pi * R^2} = \frac{A}{(6\sqrt{\pi} * V)^{\frac{2}{3}}}$
sphericity	Value calculated by the following equation; $sphericity = \frac{(6\pi^2 V)^{\frac{2}{3}}}{A}$
surface_volume_ratio	Value calculated by the following equation; $surface\_volume\_ratio = \frac{A}{V}$
Location related parameters	
MNI_str_loc.01	Occupancy rate of area "0" of the MNI structural atlas within the VOI. This area represents white matter.
MNI_str_loc.02	Occupancy rate of area "1" of the MNI structural atlas within the VOI. This area represents lateral ventricles.
MNI_str_loc.03	Occupancy rate of area "2" of the MNI structural atlas within the VOI. This area represents the cerebrum.
MNI_str_loc.04	Occupancy rate of area "3" of the MNI structural atlas within the VOI. This area represents the frontal lobe.
MNI_str_loc.05	Occupancy rate of area "4" of the MNI structural atlas within the VOI. This area represents the insular lobe.
MNI_str_loc.06	Occupancy rate of area "5" of the MNI structural atlas within the VOI. This area represents the occipital lobe.
MNI_str_loc.07	Occupancy rate of area "6" of the MNI structural atlas within the VOI. This area represents the parietal lobe.

MNI_str_loc.08	Occupancy rate of area “7” of the MNI structural atlas within the VOI. This area represents the basal ganglia.
MNI_str_loc.09	Occupancy rate of area “8” of the MNI structural atlas within the VOI. This area represents the temporal lobe.
MNI_str_loc.10	Occupancy rate of area “8” of the MNI structural atlas within the VOI. This area represents the thalamus.

1) Entropy(S) is calculated by the following equation where p_i stands for the frequency of the greyscale level i:

$$S = -\sum_{i=0}^{255} p_i \log_2 p_i$$

2) Prewitt filtering was performed by applying first order horizontal Gx and vertical Gy differentiation and by calculating the magnitude G, where Gx and Gy stand for the horizontal and vertical gradient of the image, respectively, and A for the original two-dimensional greyscale image

$$\mathrm{G}x = \begin{bmatrix} -1 & 0 & +1 \\ -1 & 0 & +1 \\ -1 & 0 & +1 \end{bmatrix} * \mathrm{A}, \quad \mathrm{G}y = \begin{bmatrix} -1 & -1 & -1 \\ 0 & 0 & 0 \\ +1 & +1 & +1 \end{bmatrix} * \mathrm{A}, \quad \mathrm{G} = \sqrt{\mathrm{G}x^2 + \mathrm{G}y^2}$$

3) The Gdzscore image was created by visualising the magnitude of enhancement calculated from both 256-leveled non-enhanced and Gadolinium-enhanced T1-weighted images; 256-leveled Gadolinium-enhanced T1-weighted images were plotted as a function of 256-leveled non-enhanced T1-weighted images in the whole brain. Linear regression fitting was applied to the data obtained, which can be expressed as follows:

$$(GdT1WI) = \alpha(T1WI) + \beta$$

where (*GdT1WI*) and (*T1WI*) are 256-leveled Gadolinium-enhanced and non-enhanced T1-weighted images. By solving α and β, one can determine the linear correlation of the 256-leveled Gadolinium-enhanced and non-enhanced T1-weighted images.
Next, the magnitude of deviation from the above solved linear regression line for any particular voxel (i) can be expressed as follows:

$$deviation_i = \frac{(GdT1WI)_i - \alpha(T1WI)_i - \beta}{\sqrt{\alpha^2 + 1}}$$

where $(GdT1WI)_i$ and $(T1WI)_i$ are the 256-leved values of voxel (i) in Gadolinium-enhanced and non-enhanced T1-weighted images.
Finally, the Gdzscore of each data point was defined as follows:

$$Gdzscore_i = \frac{deviation_i - \mu}{\rho}$$

where μ and σ are the means and standard deviation of $deviation_i$ in the whole brain.
